# Pharmacokinetics and anti-tumour activity of LM985 in mice bearing transplantable adenocarcinomas of the colon.

**DOI:** 10.1038/bjc.1986.214

**Published:** 1986-10

**Authors:** J. A. Double, M. C. Bibby, P. M. Loadman

## Abstract

LM985 is one of a series of compounds based on the flavone ring structure and selected for clinical trial primarily for its activity in colon 38 as part of the NCI screen. We have investigated the anti-tumour activity against three differing transplantable adenocarcinomas of the mouse colon (MAC). Single i.p. injection at maximum tolerated dose showed no activity against the ascitic tumour MAC 15A, moderate activity against subcutaneous tumours MAC 13 and MAC 15A and produced a significant growth delay against MAC 26. These responses against s.c. tumours were considerably enhanced by repeated injection 7 days later when greater than 90% tumour inhibition was seen in MAC 13 and cures were achieved in MAC 26. Pharmacokinetic studies confirm the rapid degradation of LM985 to LM975, the possible active principle. Analysis of area under the curve for LM975 indicated a good relationship with administered doses of LM985 and tumour responses. The MAC system shows a good correlation with human large bowel cancer and these preliminary observations with LM985 would suggest that it or its metabolite LM975 may have a value in the management of large bowel cancer.


*Br. J. Cancer* (1986), **54**, 595–600

### Pharmacokinetics and anti-tumour activity of LM985 in mice bearing transplantable adenocarcinomas of the colon

J.A. Double, M.C. Bibby & P.M. Loadman

*Clinical Oncology Unit, University of Bradford, Bradford BD7 1DP, UK.*

**Summary** LM985 is one of a series of compounds based on the flavone ring structure and selected for clinical trial primarily for its activity in colon 38 as part of the NCI screen. We have investigated the anti-tumour activity against three differing transplantable adenocarcinomas of the mouse colon (MAC). Single i.p. injection at maximum tolerated dose showed no activity against the ascitic tumour MAC 15A, moderate activity against subcutaneous tumours MAC 13 and MAC 15A and produced a significant growth delay against MAC 26. These responses against s.c. tumours were considerably enhanced by repeated injection 7 days later when >90% tumour inhibition was seen in MAC 13 and cures were achieved in MAC 26. Pharmacokinetic studies confirm the rapid degradation of LM985 to LM975, the possible active principle. Analysis of area under the curve for LM975 indicated a good relationship with administered doses of LM985 and tumour responses. The MAC system shows a good correlation with human large bowel cancer and these preliminary observations with LM985 would suggest that it or its metabolite LM975 may have a value in the management of large bowel cancer.

Oxo-4 phenyl-2,4,H-1 Benzopyranne-8-yl-acetate of dimethylamino-2-ethyl, chlorhydrate (LM985) is the ester of flavone acetic acid and dimethylethanolamine which was submitted to the NCI for pre-clinical screening in 1980. It was selected for clinical trial largely for its activity against colon 38.

Animal toxicology indicated that LM985 did not cause myelosuppression, nausea or vomiting or major organ toxicity. Lethal doses caused clonic spasms, stupor and death (Kerr *et al.*, personal communication). The mechanism of action is not known but flavonoids are capable of binding to DNA and a variety of intracellular enzymes bearing metal ions (Havsteen, 1983). Kerr *et al.* (1985) have suggested that the hydrolysis product LM975 may be the active principle (Figure 1).

The mouse adenocarcinoma of the colon (MAC) series of transplantable tumours (Double *et al.*, 1975) has been used in a variety of chemotherapy studies. Histologically the MAC tumours are similar to human colon cancer (Cowen *et al.*, 1980) and Double & Ball (1975) have demonstrated that the spectrum of chemotherapeutic sensitivity showed good correlation with the response rates of standard therapeutic agents against large bowel cancer. Definite anti-tumour activity is seen only at dose levels close to the maximum tolerated dose indicating the general insensitivity of the tumour system.

The present study investigates the anti-tumour activity of LM985 against three tumour lines with different histology, growth characteristics and spectra of chemosensitivity to standard agents and relates anti-tumour response to pharmacokinetics.

**Figure 1** Structural formulae of (A) LM985 and (B) LM975.

### Materials and methods

### *Animals*

Pure strain NMRI mice (age 6–8 weeks) from our inbred colony were used. They were fed on CRM diet (Labsure, England) and water *ad libitum*.

### *Tumour system*

The development of several adenocarcinomata of the large bowel in mice from primary tumours induced by prolonged administration of 1,2-dimethylhydrazine has been described elsewhere

Correspondence: J.A. Double
Received 7 May 1986; and in revised form 25 June 1986.

© The Macmillan Press Ltd., 1986

(Double et al., 1975). MAC 13 tumours were transplanted into female mice and MAC 26 tumours into male mice, by s.c. implantation of tumour fragments ($\sim 1 \times 2$ mm) in the flank. MAC 15A ascites tumours were transplanted into male mice by i.p. inoculation of $1 \times 10^6$ tumour cells in 0.2 ml physiological saline. This inoculation gives a survival time of ~14 days. MAC 15A cells were also inoculated s.c. into further groups of mice.

*Test compounds*

LM985 was received from the NCI via the EORTC Screening and Pharmacology Group and further supplies via Dr W.R. Vezin, CRC Formulation Unit, University of Strathclyde. LM975 was a gift from Lipha Lyonnaise Industrielle Pharmaceutique, Lyons via Professor S.B. Kaye, University of Glasgow. Positive control compounds methyl-CCNU and cyclophosphamide were gifts from the NCI and Boehringer, UK respectively. LM985 and cyclophosphamide were dissolved in physiological saline and Methyl-CCNU in 10% ethanol/arachis oil at an appropriate concentration, for a desired dose to be administered in 0.1 ml/10 g body weight. All injections were i.p.

*Chemotherapy*

Animals bearing s.c. tumours were allocated by restricted randomisation into groups of 10 and those bearing MAC 15A ascites tumours were allocated into groups of 5. With the more rapidly growing MAC 13 and 15A tumours, chemotherapy commenced 2 days after implantation. MAC 13 and MAC 15A s.c. tumours are palpable at this stage and anti-tumour responses were assessed 14 days later by recording tumour weights. MAC 15A i.p. tumours were assessed from median survival times (MST) (Geran et al., 1972). With the slower growing MAC 26 tumours chemotherapy did not commence until tumours could be reliably measured i.e. until they achieved minimum tumour volumes of 40 cm³. MAC 26 was assessed by twice weekly two-dimensional caliper measurements of the tumour. Tumour volume was calculated from the formula $a^2 \times b/2$ where $a$ is the smaller diameter and $b$ is the larger (Geran et al., 1972). Tumour volumes were normalised with respect to starting volumes and graphs of relative tumour volume against time were plotted on semi-log graph paper.

Activity scores of LM985 and positive control compounds against each tumour line were allocated as shown in Table I.

**Table I** System for scoring anti-tumour activity

| *Solid s.c. tumours* | | *MAC 15A (ascitic tumour)* | |
|---|---|---|---|
| *% inhibition*[a] | *Score* | *T/C%*[b] | *Score* |
| <25 | 0 | <125 | 0 |
| 25–49 | 1+ | 125–149 | ± |
| 50–74 | 2+ | 150–200 | 1+ |
| 75–90 | 3+ | >200 | 2+ |
| >90 | 4+ | <50% cures | 3+ |
| | | >50% cures | 4+ |

[a]Solid tumours assessed by weight or volume inhibition; [b]MST of treated animals over MST of control animals ×100.

*Reagents*

Spectroscopic grade ethanol (BDH Chemicals, Poole, Dorset), p-dimethylaminobenzaldehyde (Sigma Chemical Co., Poole, Dorset) and triple distilled water were used. Other reagents were of analytical grade. Time expired blood was obtained from the Haematology Department, Bradford Royal Infirmary. The blood was centrifuged at 2000 g for 15 min and the separated plasma stored at −20°C.

*Sample extraction and analysis*

Blood samples from three mice at each time point were taken by cardiac puncture under ether anaesthesia and collected into heparinised tubes and centrifuged at 2000 g and 4°C for 10 min. The plasma was frozen and stored at −20°C until analysis.

LM985 and LM975 were extracted from plasma using solid phase chromatography. Plasma samples (50 μl) were mixed with 0.5 ml sodium acetate-acetic acid buffer (0.1 M, pH4.0) (acetate buffer) and the internal standard (p-dimethylaminobenzaldehyde) added (100 μl at 100 μg ml$^{-1}$). Bond–Elut cartridges containing particles of $C_{18}$-coated silica (Analytichem International) were activated by passing ethanol (1 ml) then acetate buffer (1 ml) through under negative pressure using the Vac–Elut system (Analytichem International). Plasma specimens were applied to the Bond–Elut cartridges. The cartridges were then washed with buffer and air dried. Ethanol (400 μl) was passed through the cartridges and the elutents collected in tapered plastic tubes.

*Chromatography*

LM985 was measured in plasma by an HPLC method described by Kerr et al. (1985). A Lichrosorb RP-8 column was used (Merck/BDH, Poole, Dorset) with a $C_8$ pre column. An isocratic mobile phase of 12.5% methanol, 12.5% acetonitrile, 12.5% isopropanol, 62.5% 0.005 M phosphoric acid was pumped at a constant flow rate of 1.5 ml min$^{-1}$ using a Waters (Waters

Associates, Norwich, UK) 6000A pump. An ion-pair reagent, tetrabutylammonium-phosphate (Sigma), was added ($1.5\,mmol\,l^{-1}$). This both improved chromatography and reduced retention times from 21 min to 3.3 min (LM985). The injection volume was $10\,\mu l$ and detection was at 303 nm using a Waters Lambda–Max 480 LC spectrophotometer.

Two standard curves were prepared by the addition of LM985 and LM975 to control human plasma and plotting ratios of peak areas of the two compounds to the internal standard against drug concentration. This procedure was carried out in human plasma as it was not possible to detect LM985 in mouse plasma (see **Results**) but control mouse plasma was analysed and no interfering peaks were found. Peaks were traced and integrated with an Isaac Model 42A data module (Cyborg Corporation, USA), an Apple IIE computer (Apple Computers Inc., USA) and Appligration II software (Dynamic Solutions Corporation, USA). The curves were linear over the range $0.1–40\,\mu g\,ml^{-1}$. The assay was sensitive to drug concentration at $10\,ng\,ml^{-1}$. The concentration of LM985 and its metabolite were determined from their peak area ratios to the internal standard. Recovery was >90% and the coefficient of variation for replicate samples at a concentration of $5\,\mu g\,ml^{-1}$ was 6.4%. Confirmation of identity of LM975 in mouse plasma was achieved by measuring retention characteristics using two further column packing materials viz. Hypersil $C_{18}$ octadecyl-bonded silica 250 × 4.6 mm (Shandon Southern Instruments, Cheshire, UK) and SAS Hypersil methyl-bonded silica 100 × 4.6 mm (Shandon Southern).

### In vitro *stability studies*

Human plasma used in these studies was from our stock plasma stored at −20°C. Two millilitres of plasma with a plasma concentration of $1\,mg\,ml^{-1}$ LM985 (equivalent to peak plasma levels in the mouse of LM975 after 1 h) was incubated at 37°C, $50\,\mu l$ samples were taken and $900\,\mu l$ acetate buffer and $100\,\mu l$ of internal standard were immediately added to stop the reaction. LM985 and LM975 were then extracted using the Bond–Elut cartridges and $10\,\mu l$ of the eluent injected into the HPLC. Similar stability studies with LM985 in mouse plasma and whole blood showed immediate hydrolysis of LM985 to LM975. LM985 was shown to be stable in mouse plasma at an acid pH.

### *Pharmacokinetic analysis*

The area under the plasma concentration versus time curve (AUC) was calculated using the trapezoid rule.

### Results

LM985 had no effect against MAC 15A grown as an ascites tumour (Table II). When MAC 15A cells were grown subcutaneously significant anti-tumour effects were seen (Table III). Chemosensitivity of established tumours treated on day 9 was significantly greater than those treated on day 2. Single dose LM985 above $200\,mg\,kg^{-1}$ showed only moderate activity against MAC 13 s.c. tumours whereas responses were considerably enhanced by repeated injection 7 days later (Table IV). Maximum tolerated dose ($400\,mg\,kg^{-1}$) produced a significant growth delay against the well-differentiated slower growing MAC 26 tumours (Figure 2) and repeated injection at this dose level 7 days later produced cures.

Pharmacokinetic studies indicate a rapid degradation of LM985 to LM975 in human plasma at 37°C *in vitro* (Figure 3). Similar studies with

**Table II** Activity of LM985 against MAC 15A i.p.

| *Dose ($mg\,kg^{-1}$)* | | | | |
|---|---|---|---|---|
| *Day 2* | *Day 9* | *Vehicle* | *T/C%* | *Activity* |
| 800 | — | 0.9% saline | 23 | toxic |
| 400 | 400 | 0.9% saline | 71 | toxic |
| 400 | — | 0.9% saline | 107 | 0 |
| 200 | 200 | 0.9% saline | 111 | 0 |
| 200 | — | 0.9% saline | 92 | 0 |
| 100 | 100 | 0.9% saline | 104 | 0 |
| 100 | — | 0.9% saline | 100 | 0 |
| Positive control MethylCCNU 20 | — | Ethanol/ arachis oil (1/10) | 154 | 1+ |
| Control | — | — | — | |

**Table III** Activity of LM985 against MAC 15A s.c.

| *Dose (mg kg⁻¹)* | | | | | |
|---|---|---|---|---|---|
| *Day 2* | *Day 9* | *Vehicle* | *Survivors* | *T/C%* | *Activity* |
| 400 | — | 0.9% saline | 10/10 | 51 | 1+ |
| 400 | 400 | 0.9% saline | 5/10 | 14 | toxic |
| — | 400 | 0.9% saline | 10/10 | 18 | 3+ |
| Control | — | — | 10/10 | — | — |

**Table IV** Activity of LM985 against MAC 13

| *Dose (mg kg⁻¹)* | | | | | |
|---|---|---|---|---|---|
| *Day 2* | *Day 9* | *Vehicle* | *Survivors* | *T/C%* | *Activity* |
| 800 | — | 0.9% saline | 0/10 | — | toxic |
| 400 | 400 | 0.9% saline | 9/10 | 9 | 4+ |
| 400 | — | 0.9% saline | 10/10 | 40 | 2+ |
| — | 400 | 0.9% saline | 10/10 | 28 | 2+ |
| 200 | 200 | 0.9% saline | 10/10 | 45 | 2+ |
| 200 | — | 0.9% saline | 10/10 | 78 | 0 |
| 100 | 100 | 0.9% saline | 10/10 | 72 | 1+ |
| 100 | — | 0.9% saline | 10/10 | 68 | 1+ |
| Positive control | | Ethanol/ | | | |
| MethylCCNU | | arachis oil | 10/10 | 4 | 4+ |
| 20 | — | (1/10) | | | |
| Control | — | — | 10/10 | — | |

**Figure 2** Activity of LM985 against MAC 26. (○—○ untreated control; ●—● LM985 200 mg kg⁻¹ day 0, day 7, activity score 1+; ■—■ LM985 400 mg kg⁻¹ day 0, activity score 3+; ▲—▲ LM985 400 mg kg⁻¹ day 0, day 7, activity score 4+.

**Figure 3** Breakdown of LM985 to LM975 in human plasma at 37°C *in vitro* at a concentration of 1 mg ml⁻¹ LM985.

mouse plasma and whole blood indicated immediate breakdown of LM985 to LM975, with no detection of the former compound, despite extraction procedures commencing within 20 sec of its addition to the plasma.

Analysis of mouse plasma following 3 *in vivo* dose levels of LM985 indicates a dose relationship between AUC's for LM975 and the adminitered dose of LM985 (Figure 4).

**Figure 4** Mouse plasma levels (±s.d.) of LM975 following i.p. injection of different *in vivo* dose levels of LM985. ▲—▲ 100 mg kg$^{-1}$ AUC 0.95±0.26 (s.e.) mg h ml$^{-1}$, ●—● 200 mg kg$^{-1}$ AUC 2.1±0.31 mg h ml$^{-1}$, ■—■ 400 mg kg$^{-1}$ AUC 3.89±0.54 mg h ml$^{-1}$.

### Discussion

This investigation demonstrates that LM985 has significant anti-tumour activity in a panel of transplantable mouse colon tumours in mice thus confirming the activity previously seen in colon 38. Single i.p. injection at maximum tolerated dose showed no activity against ascitic MAC 15A, moderate activity against MAC 13 and subcutaneous MAC 15A and produced a significant growth delay against MAC 26. These latter responses were considerably enhanced by repeated injection 7 days later when greater than 90% tumour inhibition was seen in MAC 13 and cures were seen in MAC 26. The activity (4+) against MAC 26 is particularly impressive as the previous best recorded response with this tumour line seen with cyclophosphamide is 3+ i.e. a growth delay of approximately 9 days or 80% tumour inhibition. Cures have not previously been achieved in this tumour line.

This observation perhaps gives an insight to factors involved in tumour responses. By nature of the treatment protocol employed for the MAC 26 line, tumours are well established with a good vascular supply. Our basic screening protocol with MAC 13 uses 2 day old tumours which will not be as well established, whereas in the repeated treatment protocol the tumours although smaller than the controls were well established. Good responses were also seen with tumours treated on day 9 only, suggesting that the establishment of a blood vascular system may be an important factor in tumour responses to LM985.

Kerr *et al.* (1985) have suggested that the hydrolysis product flavone acetic acid (LM975) may in fact be the active principle of this agent. If this product is produced in the serum it is unlikely that an ascitic tumour would show any significant level of response and this is in fact the case with MAC 15A. Subsequent transplantation of this tumour line in a subcutaneous site revealed that these cells were sensitive.

Pharmacokinetic studies have confirmed the rapid degradation of LM985 to LM975 in human plasma *in vitro*. Similar studies with mouse plasma and whole blood have indicated immediate breakdown of LM985 to LM975 *in vitro*. Analysis of mouse plasma following different *in vivo* dose levels of LM985 indicated a good dose relationship between levels of LM975 and the administered dose of LM985. Area under the curve values of LM975 show a good relationship with anti-tumour response suggesting LM975 to be the active principle. A phase I trial with LM985 has been completed and results indicated that dose limiting toxicity was acute reversible hypotension (Kerr *et al.*, personal communication). These authors recommend that LM975 be considered for clinical trial as they suggest that substantially higher doses of LM975 can be given without dose limiting cardiovascular toxicity. The observations in this study would indicate that LM975 may be of value in the management of cancer as the MAC system has been shown to be a good model of human disease with responses to standard agents only seen close to maximum tolerated dose. These flavonoid compounds are novel structures which may have completely different mechanisms of action from conventional agents and investigations are currently being undertaken to determine factors involved in their antitumour activity.

The authors acknowledge Professor A.F. Fell and Dr B.J. Clark, School of Pharmaceutical Chemistry, University of Bradford for providing further proof of the identity of LM975.

The work was supported by the White Watson/Turner Cancer Research Trust, Bradford.

### References

COWEN, D.M. DOUBLE, J.A. & COWEN, P.N. (1980). Some biological characteristics of transplantable lines of mouse adenocarcinoma of the colon. *J. Natl Cancer Inst.*, **64,** 675.

DOUBLE, J.A. & BALL, C.R. (1975). Chemotherapy of transplantable adenocarcinoma of the colon in mice. *Cancer Chemother. Rep.*, **59,** 1083.

DOUBLE, J.A., BALL, C.R. & COWEN, P.N. (1975). Transplantation of adenocarcinoma of the colon in mice. *J. Natl Cancer Inst.*, **54,** 271.

GERAN, R.I., GREENBERG, N.H., MACDONALD, M.M., SCHUMACHER, A.M. & ABBOT, B.J. (1972). Protocols for screening chemical agents and natural products against tumours and other biological systems (third edition). *Cancer Chemother. Rep.*, **3,** 1.

HAVSTEEN, B. (1983). Flavonoids, a class of natural products of high pharmacological potency. *Biochem. Pharmacol.*, **32,** 1141.

KERR, D.J., KAYE, S.B., CASSIDY, J. & 6 others. (1985). A clinical pharmacokinetic study of LM985 and LM975. *Br. J. Cancer*, **52,** 467.


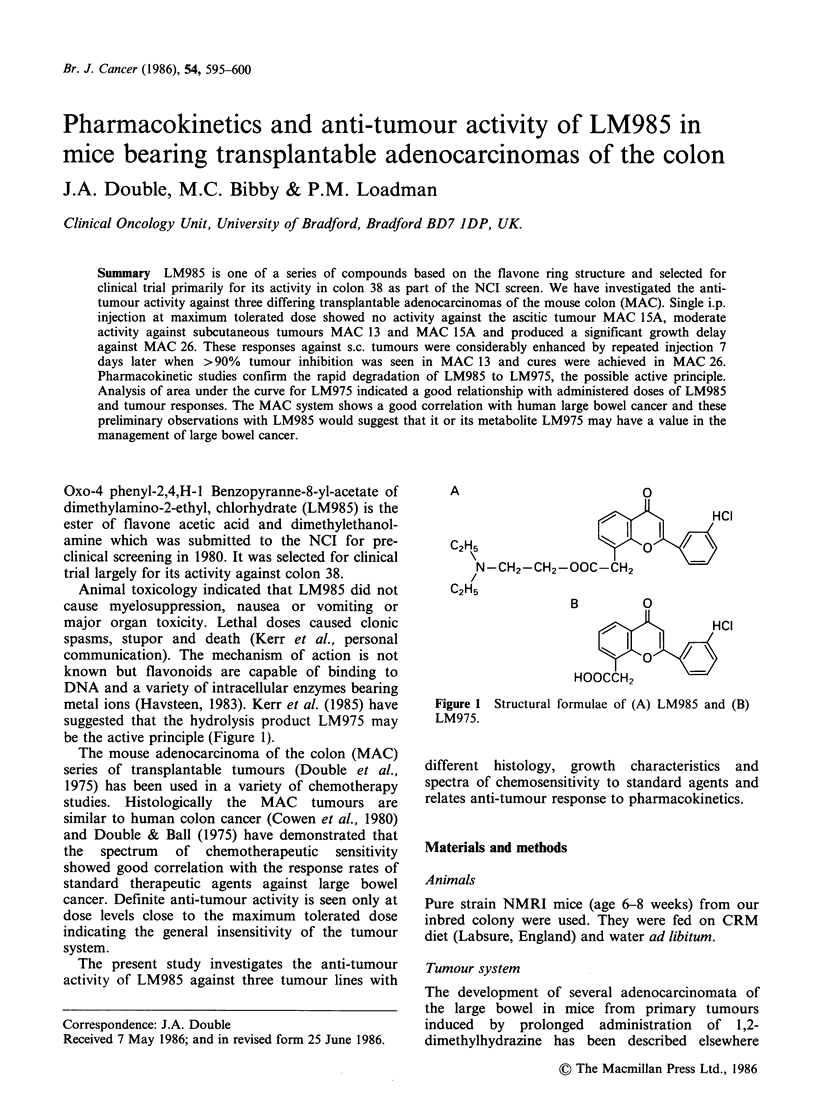

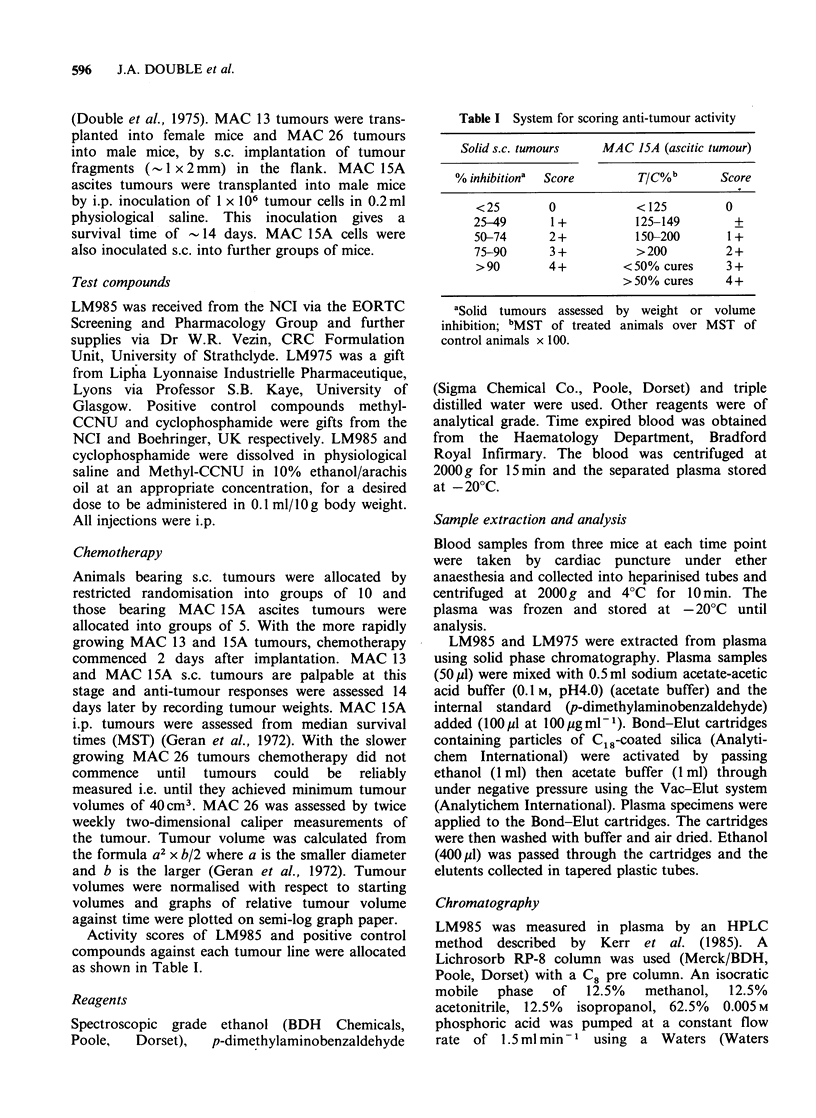

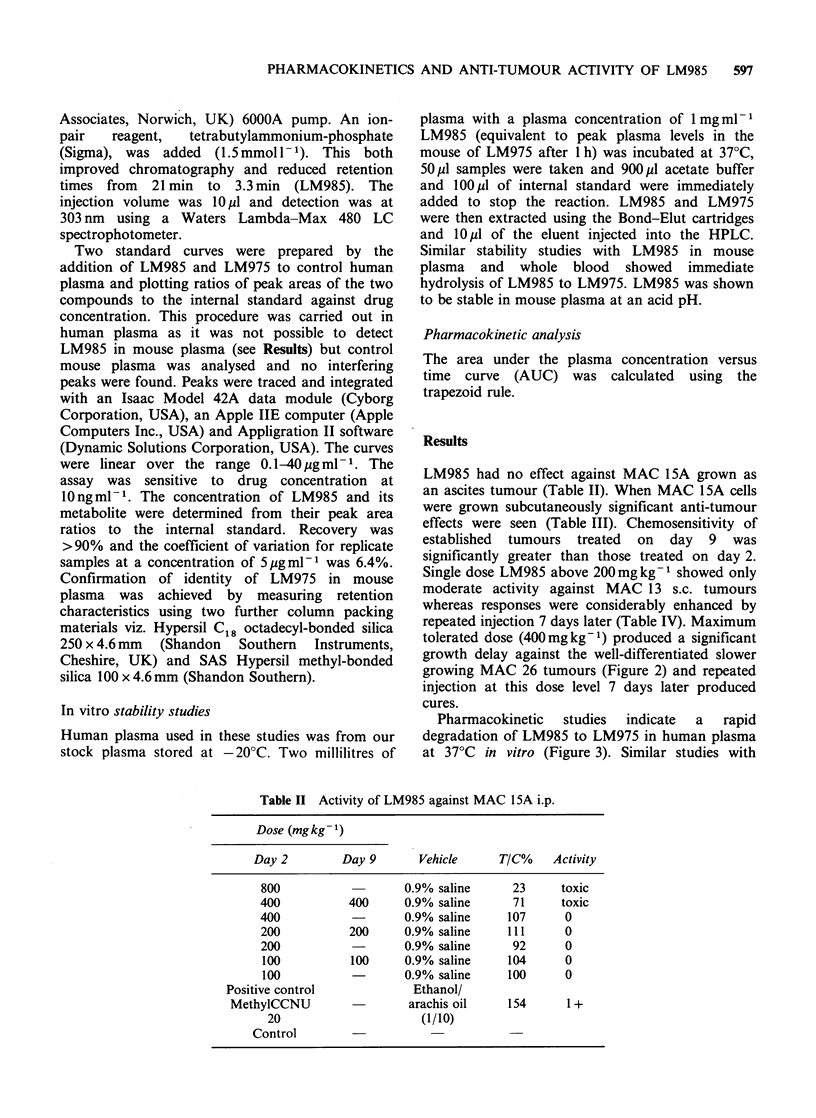

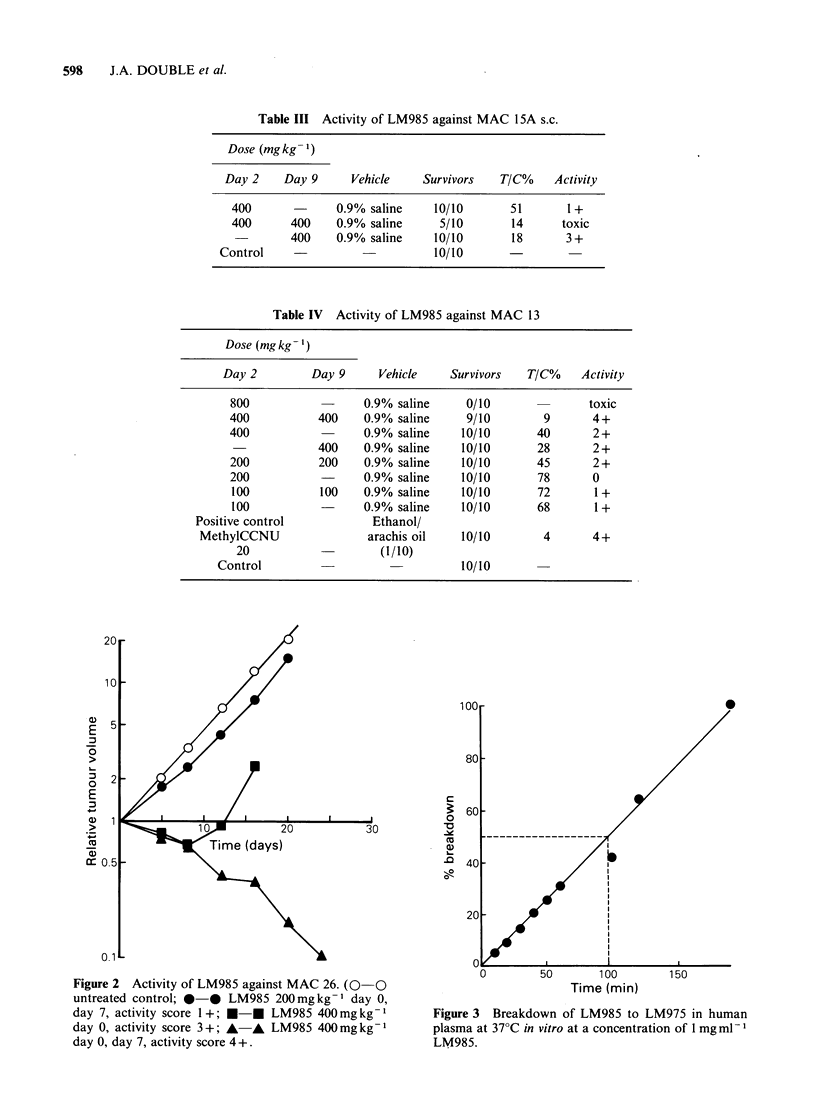

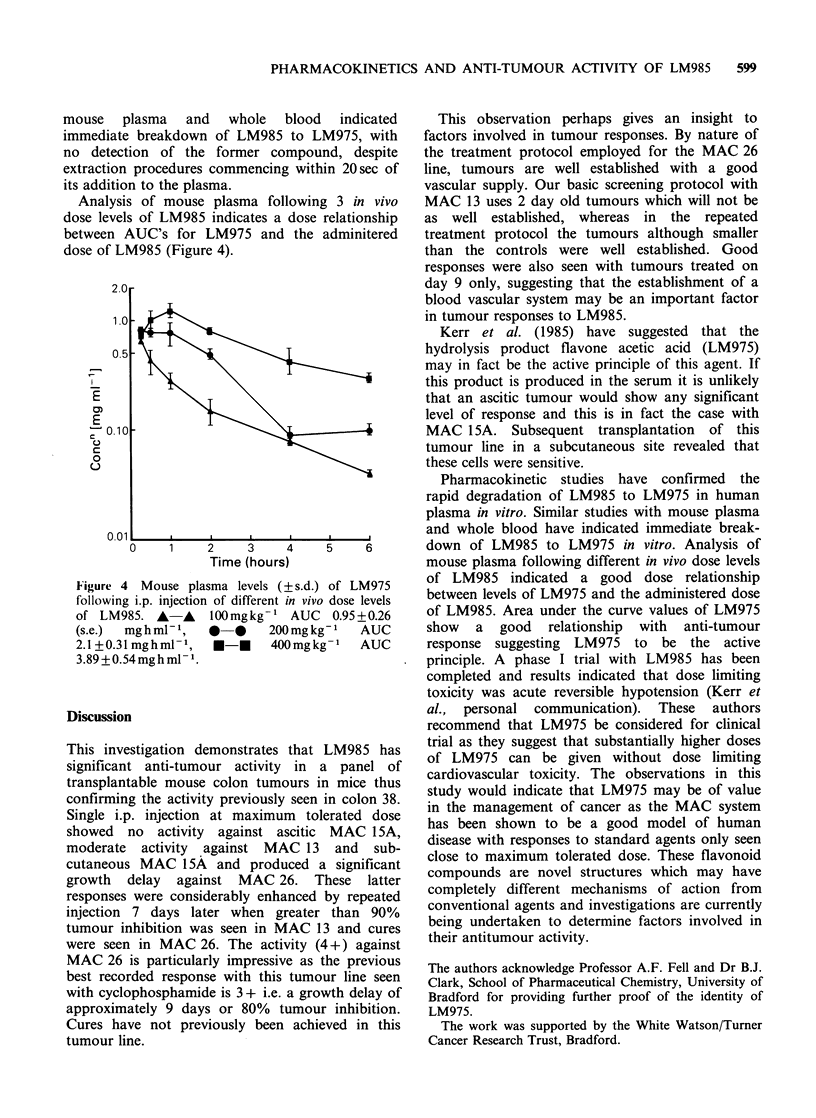

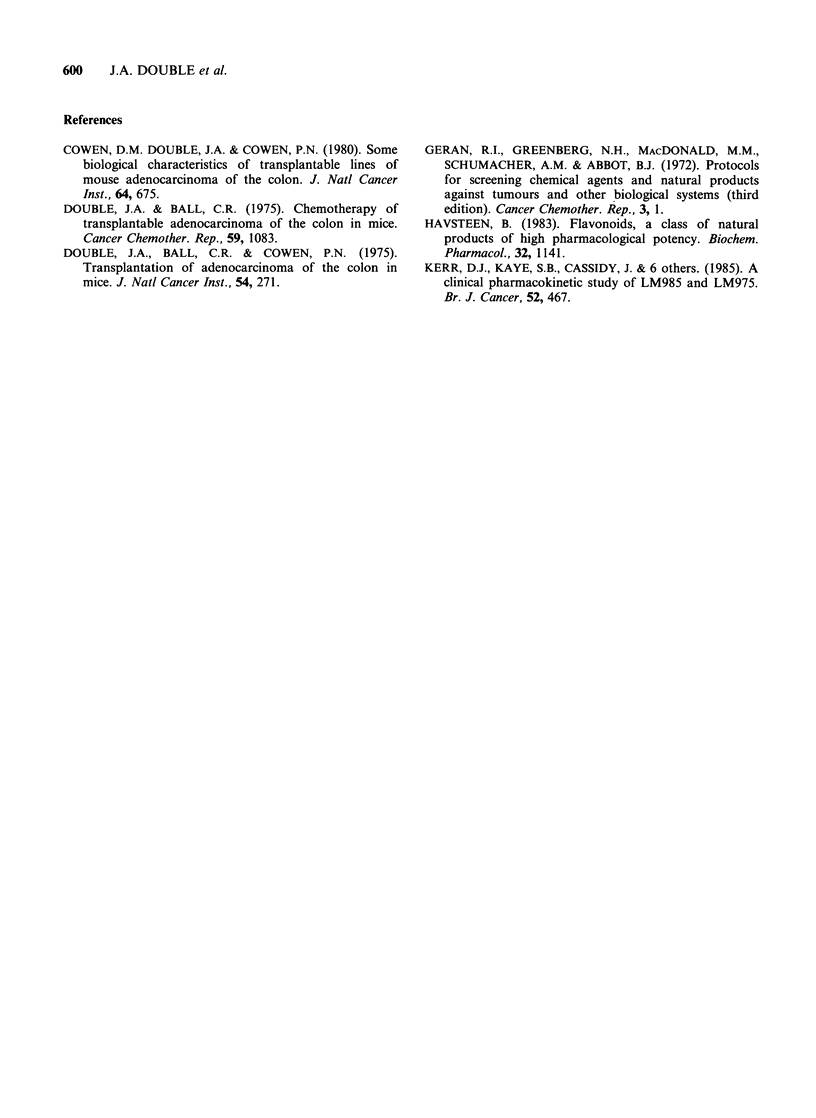


## References

[OCR_00523] Cowen D. M., Double J. A., Cowen P. N. (1980). Some biological characteristics of transplantable lines of mouse adenocarcinomas of the colon.. J Natl Cancer Inst.

[OCR_00529] Double J. A., Ball C. R. (1975). Chemotherapy of transplanted adenocarcinomas of the colon in mice.. Cancer Chemother Rep.

[OCR_00534] Double J. A., Ball C. R., Cowen P. N. (1975). Transplantation of adenocarcinomas of the colon in mice.. J Natl Cancer Inst.

[OCR_00546] Havsteen B. (1983). Flavonoids, a class of natural products of high pharmacological potency.. Biochem Pharmacol.

